# Tumor Predisposing Post-Zygotic Chromosomal Alterations in Bladder Cancer—Insights from Histologically Normal Urothelium

**DOI:** 10.3390/cancers16050961

**Published:** 2024-02-27

**Authors:** Wiktoria Stańkowska, Daniil Sarkisyan, Bożena Bruhn-Olszewska, Katarzyna Duzowska, Michał Bieńkowski, Marcin Jąkalski, Magdalena Wójcik-Zalewska, Hanna Davies, Kinga Drężek-Chyła, Rafał Pęksa, Agnieszka Harazin-Lechowska, Aleksandra Ambicka, Marcin Przewoźnik, Agnieszka Adamczyk, Karol Sasim, Wojciech Makarewicz, Marcin Matuszewski, Wojciech Biernat, Josef D. Järhult, Miklós Lipcsey, Michael Hultström, Robert Frithiof, Janusz Jaszczyński, Janusz Ryś, Giulio Genovese, Arkadiusz Piotrowski, Natalia Filipowicz, Jan P. Dumanski

**Affiliations:** 1 3P-Medicine Laboratory, Medical University of Gdańsk, M. Sklodowskiej-Curie 3A, 80-210 Gdańsk, Poland; wiktoria.stankowska@gumed.edu.pl (W.S.); katarzyna.duzowska@gumed.edu.pl (K.D.); marcin.jakalski@gumed.edu.pl (M.J.); magdalena.wojcik@gumed.edu.pl (M.W.-Z.); kinga.drezek-chyla@gumed.edu.pl (K.D.-C.); arkadiusz.piotrowski@gumed.edu.pl (A.P.); 2 Department of Immunology, Genetics and Pathology and Science for Life Laboratory, Uppsala University, BMC, Husargatan 3, 751 08 Uppsala, Sweden; daniil.sarkisyan@igp.uu.se (D.S.); bozena.bruhn-olszewska@igp.uu.se (B.B.-O.); hanna.davies@igp.uu.se (H.D.); 3 Department of Pathomorphology, Medical University of Gdańsk, M. Sklodowskiej-Curie 3A, 80-210 Gdańsk, Poland; michal.bienkowski@gumed.edu.pl (M.B.); rafal.peksa@gumed.edu.pl (R.P.); wojciech.biernat@gumed.edu.pl (W.B.); 4 Department of Tumor Pathology, Maria Skłodowska-Curie National Research Institute of Oncology, Garncarska 11, 31-115 Kraków, Poland; agnieszka.harazin@krakow.nio.gov.pl (A.H.-L.); marcin.przewoznik@krakow.nio.gov.pl (M.P.); agnieszka.adamczyk@onkologia.krakow.pl (A.A.); janusz.rys@krakow.nio.gov.pl (J.R.); 5 Clinic of Urology and Oncological Urology, Specialist Hospital of Kościerzyna, Piechowskiego 36, 83-400 Kościerzyna, Poland; karsas@o2.pl; 6 Clinic of General and Oncological Surgery, Specialist Hospital of Kościerzyna, Piechowskiego 36, 83-400 Kościerzyna, Poland; wojmakar@wp.pl; 7 Department and Clinic of Urology, Medical University of Gdańsk, M. Sklodowskiej-Curie 3A, 80-210 Gdańsk, Poland; marcin.matuszewski@gumed.edu.pl; 8 Zoonosis Science Center, Department of Medical Sciences, Uppsala University, Akademiska Sjukhuset, 751 85 Uppsala, Sweden; josef.jarhult@medsci.uu.se; 9 Department of Surgical Sciences, Anesthesiology and Intensive Care, Uppsala University, Akademiska Sjukhuset, 751 85 Uppsala, Sweden; miklos.lipcsey@uu.se (M.L.); michael.hultstrom@mcb.uu.se (M.H.); robert.frithiof@uu.se (R.F.); 10 Hedenstierna Laboratory, Department of Surgical Sciences, Uppsala University, Akademiska Sjukhuset, 751 85 Uppsala, Sweden; 11 Integrative Physiology, Department of Medical Cell Biology, Uppsala University, BMC, Husargatan 3, 751 08 Uppsala, Sweden; 12 Department of Urology, Maria Skłodowska-Curie National Research Institute of Oncology, Garncarska 11, 31-115 Kraków, Poland; jtjmed@interia.pl; 13 Department of Genetics, Harvard Medical School, 77 Avenue Louis Pasteur, Boston, MA 02115, USA; giulio@broadinstitute.org; 14 Department of Biology and Pharmaceutical Botany, Medical University of Gdańsk, Hallera 107, 80-416 Gdańsk, Poland

**Keywords:** bladder carcinoma (BLCA), cystectomy, transurethral resection of bladder tumor (TURBT), post-zygotic mutations, normal urothelium, chromosomal copy number alterations, loss of heterozygosity (LOH), copy neutral loss of heterozygosity (CN-LOH), mosaic loss of chromosome Y (LOY)

## Abstract

**Simple Summary:**

We were motivated by the need to improve early detection of bladder cancer (BLCA). Our aim was to uncover genetic alterations within the bladder’s lining, called urothelium, where BLCA starts. We examined normal tissue margins near and far away from tumors, studying the abnormalities that may predispose to BLCA. Recognizing the post-zygotic variability in bladder urothelium resulting from exposure to carcinogens in urine, our novel approach examined up to eight fragments of normal epithelium from each individual. Using a sensitive computational method, we successfully identified novel, but also well-known cancer-related regions across normal samples, confirming the efficacy of our methodology. Additionally, we provide further evidence for the already recognized connection between the loss of chromosome Y in men and BLCA. This positions our method for broader use in larger cohorts and different cancer types. Looking ahead, our methodology holds promise for identifying biomarkers that predict BLCA in analysis of urine.

**Abstract:**

Bladder urothelial carcinoma (BLCA) is the 10th most common cancer with a low survival rate and strong male bias. We studied the field cancerization in BLCA using multi-sample- and multi-tissue-per-patient protocol for sensitive detection of autosomal post-zygotic chromosomal alterations and loss of chromosome Y (LOY). We analysed 277 samples of histologically normal urothelium, 145 tumors and 63 blood samples from 52 males and 15 females, using the in-house adapted Mosaic Chromosomal Alterations (MoChA) pipeline. This approach allows identification of the early aberrations in urothelium from BLCA patients. Overall, 45% of patients exhibited at least one alteration in at least one normal urothelium sample. Recurrence analysis resulted in 16 hotspots composed of either gains and copy number neutral loss of heterozygosity (CN-LOH) or deletions and CN-LOH, encompassing well-known and new BLCA cancer driver genes. Conservative assessment of LOY showed 29%, 27% and 18% of LOY-cells in tumors, blood and normal urothelium, respectively. We provide a proof of principle that our approach can characterize the earliest alterations preconditioning normal urothelium to BLCA development. Frequent LOY in blood and urothelium-derived tissues suggest its involvement in BLCA.

## 1. Introduction

Bladder cancer (BLCA) is the most common malignancy affecting the urinary tract and has the highest incidence in higher-income countries [1]. Muscle-invasive BLCA can metastasize and is associated with a median survival of ~15 months [2]. However, BLCA detected early, before the muscle invasive stage is developed, can be treated very efficiently. Despite the improvement of survival rates for many common cancers, five-years survival rate for BLCA remains low, around 70% in Europe and the US (https://gco.iarc.fr/today accessed on 23 November 2023). Tobacco smoking and occupational exposure to various chemicals, in addition to genetic predisposition, are risk factors for BLCA, with smoking emerging as the most prominent risk, accounting for approximately 50% of all cases [1].

BLCA also shows a strong male bias and the discrepancy between sexes exists irrespective of age at diagnosis [3,4,5]. About 80% of BLCA occur in males, which suggests male-sex specific predisposition and hematopoietic loss of chromosome Y (LOY) is a good candidate for such a factor [6]. Indeed, recent evidence indicates LOY as a causative event in BLCA via dysfunction of the adaptive immune system in a mouse model [7]. Smoking has previously been shown to induce LOY [8] and this represents another link between BLCA, LOY and tobacco smoking. It is therefore not surprising that LOY has been found in 10–40% of BLCA tumors [9,10,11,12,13,14,15]. However, the relative contributions to the total LOY observed in the tumor micro-environment, which is either derived from hematopoietic cells with LOY infiltrating tumor or LOY that develops in tumor precursor cells derived from urothelium, needs to be better defined.

LOY is a common form of clonal mosaicism that occurs with high frequency in hematopoietic cells of aging men [6,16,17]. Single-cell analyses of peripheral blood mononuclear cells (PBMCs) from 29 men (median age of 80 years), revealed that every studied individual had cells with LOY, making it the most prevalent post-zygotic mutation [18]. Although LOY is more commonly found in leukocytes, it has also been detected in other tissues, albeit with lower frequencies [19]. LOY was recently proposed as a “common soil” of shared mechanisms that increase the susceptibility to genome instability and cancer in many cell types [17]. Moreover, the dysregulation of autosomal gene expression in leukocytes with LOY in a pleiotropic fashion affects many of their immune cell functions [18]. Numerous studies have also shown a strong association between hematopoietic LOY and all-cause mortality [6,20] as well as an increased prevalence or poor outcome of many diseases in males, for instance, cardiovascular disease [21,22], Alzheimer’s disease [16,23], stroke [24], COVID-19 [25] and multiple types of cancer, among them BLCA [6,26].

BLCA, originating from the urothelium, stands out among other major cancer types due to its notably high mutation rates and a diverse array of driver mutations [1]. On the other hand, the urothelium of the normal bladder is among the slowest dividing epithelia in the human body [27,28,29]. Nevertheless, this typically quiescent epithelium can undergo rapid proliferation in response to damage, due to persistent exposure to mutagens from urine, thereby increasing the likelihood of mutations and aligning with the concept of field cancerization. Indeed, a study by Lawson et al. underscored substantial diversity in mutational processes and selection dynamics within normal urothelium, which resulted in the identification of 17 genes under positive selection [30]. Another large-scale TCGA-based analysis of 27 cancer types showed post-zygotic chromosomal alterations in 18% of normal urothelium samples, adjacent to the tumor from BLCA patients [30,31]. This positions BLCA as one of the top cancers, when overall load of mutations is considered.

BLCA manifests different molecular subtypes with distinct pathogenic pathways, contingent on whether it is non-muscle invasive (NMIBC) or muscle-invasive (MIBC) cancer. As could be expected, MIBC is characterized by a higher mutational burden compared to NMIBC. The key genes such as *TP53*, *FGFR3* and *TERT* significantly contribute to BLCA initiation, progression and clinical outcomes. Furthermore, prevalent chromosome 9 deletions impact genes like *CDKN2A* in approximately 50% of both NMIBC and MIBC. In terms of genes most commonly affected by copy number changes, common deletions (*CDKN2A*, *PTEN*) and gene amplifications (*E2F3*, *MDM2*, *CCND1*, *CCNE1*, *FGFR3*, *ERBB2*, *EGFR*, *PPARG*) play roles in cell cycle regulation, RAS-MAPK and PIK3 signalling [1,32,33].

**Figure 1:**
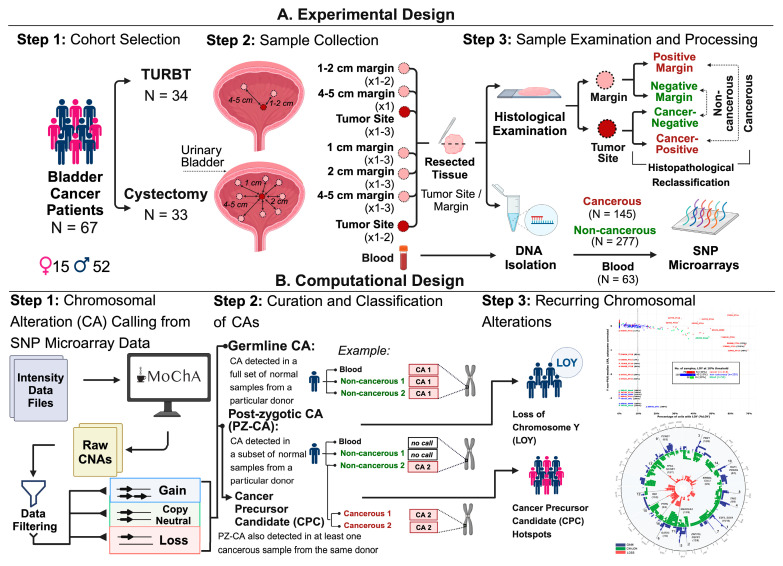
Experimental and computational workflow in the study. (**A**) From each patient (*n* = 67), a minimum of one tumor specimen and several margins at varying distances were studied. Cancer cell content was determined through histopathological examination, resulting in 145 cancerous, 277 non-cancerous- and 63 blood samples. DNA was genotyped using the Infinium Global Screening BeadChip. (**B**) Outline of the computational pipeline for detection of chromosomal aberrations in above samples. The Mosaic Chromosomal Alteration (MoChA) caller was used to detect loss, gain and CN-LOH. Alteration profiles of blood and non-cancerous samples from each patient were used to classify them into germline and post-zygotic. Alterations classified as post-zygotic in non-cancerous samples were then compared against at least one matched cancerous profile, with shared change indicating plausible autosomal “cancer precursor candidate”. These candidates were evaluated across the entire cohort, revealing 16 distinct post-zygotic alteration hotspots in non-cancerous samples. The loss of chromosome Y (LOY) was assessed across 380 samples within a subset of 52 male donors. Abbreviations: BLCA—bladder cancer; TURBT—transurethral resection of bladder tumor; CN-LOH—copy-neutral loss of heterozygosity; LOY—loss of chromosome Y; CA—chromosomal alteration; PZM—post-zygotic mutation.

In conclusion, further research is essential to enhance our understanding of the pathogenesis of BLCA, improvement of early diagnostics and clinical treatment. We aimed here at the characterization of the histologically normal urothelium to evaluate the hypothesis of field cancerization in BLCA. Towards this goal, we studied numerous normal non-cancerous urothelial margins located at varying distances from the tumor (2–8 samples per donor), along with primary tumors and peripheral blood from 67 patients diagnosed with bladder cancer (Figure 1). We defined non-cancerous samples of urothelium as proximal margin (PM) and distal margin (DM), which represent non-contiguous tissue with the tumor focus and were located 1–2 cm and 4–5 cm away from the tumor, respectively. Histopathological analysis of these samples confirmed the absence of cancer cells.

## 2. Materials and Methods

### 2.1. Patients and Samples Studied

The 67 BLCA patients for the project, who underwent either radical cystectomy or transurethral resections of bladder tumor (TURBT), with or without the preoperative chemotherapy treatment, were recruited in three large hospitals. Written informed consent was obtained from all the patients prior to surgery. All procedures were performed in accordance with the relevant national and international laws and guidelines as well as in compliance with the European Union General Data Protection Regulation (EU GDPR) and were approved by the Independent Bioethics Committee for Research at the Medical University of Gdansk (approval number NKBBN/564/2018 with multiple amendments). The collection protocols were precisely designed and are described in detail in the previous paper [34]. In case of radical cystectomy surgeries after macro-sectioning of the resected organ, small tissue fragments were selected and excised for biobanking. For TURBT patients, the fragments were collected during the surgery, using a loop of cystoscope. Subsequently, each fragment was cut in half: one portion was fresh-frozen at −80 °C, while the other one was fixed in formalin, embedded in paraffin and underwent standard processing (FFPE), sectioning and hematoxylin and eosin (H&E) staining. The latter FFPE tissue sectioning was done along the cutting surface closest to the fresh-frozen biobanked piece of tissue, so that the FFPE section is as much as possible representative for the tissue in the frozen specimen. The microscopic examination of the fragments collected for our study was conducted by a pathologist as part of the clinical routine. Using the approach of two halves of tissue fragments, we could confidently confirm the content of biobanked material that was later used for the molecular analysis. DNA extraction from bladder urothelium (input of 5–15 mg of fresh frozen fragments) was performed using standard phenol/chloroform method with several in-house modifications [34]. DNA from 200 µL of whole blood was isolated using QuickGene DNA whole blood kit S (Kurabo, Osaka, Japan) with QuickGene-Mini 480 instrument (Kurabo).

### 2.2. MoChA for Detection of Autosomal Chromosomal Alterations (ACAs)

SNP&SEQ Technology Platform, Uppsala, Sweden, part of the National Genomics Infrastructure (NGI) Sweden and Science for Life Laboratory performed genotyping for the BLCA cohort using Infinium Global Screening Array Multiethnic Disease Version 3.0 (GSAMD-24v3-0-EA_20034606). Intensity data were processed via Mosaic Chromosomal Alterations (MoChA) [35] WDL pipeline version 2022-05-18 (https://github.com/freeseek/mochawdl, accessed on 27 June 2022), according to author’s instructions and recommended quality check (QC) procedures. The output was filtered to remove samples with call rate < 0.97, BAF autocorrelation > 0.03, which is indicative of sample contamination, and sex discordance. High aberration load in the cancer samples often appeared as a formal violation of above QC thresholds. The decision to remove or keep such cancer samples was made after manual examination of hybridization quality using whole genome LRR and BAF plots. ACA calls of <10 bp were removed. Clusters of ACAs with pairwise reciprocal overlap >50% detected in WB and in all non-cancerous PM- and DM samples from the same donor were further removed as likely corresponding to a germline mutation. Following Loh et al. 2020 [35], we further removed the likely germline ACAs in non-cancerous samples: calls flagged via MoChA as inherited copy number polymorphisms, calls with an LOD score of <10 for the model based on BAF and genotype phase and calls < 500 kbp with relative coverage estimate > 2.5. The exact filtering formula is available on https://github.com/freeseek/mocha (accessed on 27 June 2022) as “Generate list of non-germline events”.

### 2.3. MoChA for Detection of Loss of Chromosome Y (LOY)

The intensity data in pseudoautosomal region 1 (PAR1) and in the non-PAR region (non-PAR) also called male specific part of chromosome Y (MSY) on sex chromosomes of GRCh37 genome were processed via MoChA to identify LOY in male subjects. GSAMD-24v3-0-EA_20034606 genotyping array had 632 SNPs in PAR1 (Y:10,001–2,649,520 was reported via MoChA as X:60,001–2,699,520), and 5224 SNPs in non-PAR (Y:2,649,521–59,034,049). Following the filtering formula “Generate list of samples with mosaic loss of chromosome Y (LOY)” from https://github.com/freeseek/mocha (accessed on 27 June 2022), male samples without >2 Mbp alteration on PAR1 were taken as having no detectable LOY. Otherwise, LOY was scored using the longest alteration on chromosome X. Five PT samples having both relative coverage estimate >2.5 and highest autosome corrected median LRRs of non-PAR regions of chromosome Y were removed from analysis as likely having either mosaic gain of chromosome Y (GOY) or XXY genotype.

### 2.4. ddPCR for LOY Detection

The LOY status was determined using ddPCR as described previously [25]. Briefly, DNA samples were pre-digested with *HindIII* (Thermo Fisher, Waltham, MA, USA). Subsequently, 50 ng of the digested DNA was used in the analysis. The digested DNA was mixed with PCR supermix for probes without dUTP (BioRad, Hercules, CA, USA) together with primers and probes for the AMELX/AMELY TaqMan-assay, number C_990000001_10 (Thermo Fisher). Quantification of the relative number of chromosomes X and Y in the sample was obtained by targeting the 6 bp sequence difference present between the AMELX and AMELY genes. Droplets were generated using the automated droplet generator (Bio-Rad), PCR was conducted using the T100 thermal cycler (Bio-Rad) and a QX200 Droplet reader (Bio-Rad) was used for the fluorescent measurements of droplets. The data were analyzed using the QuantaSoft software version 1.7.4 (Bio-Rad).

### 2.5. Statistical Analysis

Data were analyzed using Bayesian regression models with Bernoulli distribution family by calling *Stan* 2.26 [36] from *R* 4.2 [37] using the *brms* 2.20 interface [38]. Predictors were centered and scaled. Models had no intercepts with indexing approach to predictors [39]. In accordance with Stan recommendations [40], weakly informative priors were used for group-level effects, residual SD and group-level SD. *p*-values were produced by frequentist summary in *emmeans* 1.8 package [41]. Familywise Bonferroni multiple testing correction was performed for reported *p*-values. Posterior distribution medians and 95% highest posterior density credible intervals (95% HDI) were plotted. Significant contrasts between groups were reported if adjusted *p* ≤ 0.05 and 95% HDI did not contain 0. Pearson’s correlation coefficients were calculated using the *cor.test* function, and linear model was calculated using *lm*, *summary* and *confint* functions from *stats* package [37]. Levels of statistical significance were shown as follows: *** *p* < 0.001, ** *p* < 0.01, * *p* < 0.05; not significant, *p* ≥ 0.05.

## 3. Results

### 3.1. Cohort Characteristics and Histopathological Classification of Samples

Our study follows the concept of age-related clonal expansions of normal cells in tissue that give rise to solid tumors of the breast [42,43,44,45] and the model of field cancerization [46,47,48,49], which was the main rationale behind collecting multiple samples from normal margin of bladder urothelium as well as primary tumors and blood. The blood-derived DNA allows genotypic comparisons with the tissue that is not primarily involved in the initiation of tumor formation. We consecutively collected 67 patients diagnosed with bladder cancer (BLCA) between 2019 and 2021 in three clinics in Poland. Of these, 52 were males and 15 were females and the observed male bias in our cohort is representative of a general gender discrepancy of BLCA [1,3,4,5,34]. The median age of diagnosis for males and females was 69 years (range: 42–92) and 69 years (range: 52–78), respectively. Comprehensive clinical information, such as smoking habits, tumor invasiveness, grade, preoperative treatment description and other details are given in Table S1. The general outline of our study is shown in the graphical abstract (Figure 1).

The patients recruited in the project underwent either radical cystectomy or TURBT. The further inclusion conditions comprised the availability of a detailed histopathological report for all studied margins of normal urothelium and tumors, the presence of at least one cancer sample and at least two non-cancerous samples per patient, as well as no family history of bladder cancer. Additionally, via medical questionnaires, we collected information from each donor covering family history of cancer, chronic illnesses and smoking habits. The final study cohort comprised 33 patients treated with cystectomy and 34 who underwent TURBT. The procedures for sample collection adhered to the well-established multi-sample- and multi-tissue-per-patient protocol [34]. Tissues were collected from primary tumor (PT) or tumors in the case of multifocal disease, proximal margins (PMs), distal margins (DMs) and whole blood samples (WB). The number of solid tissue samples collected for each donor varied depending on the type of surgical procedure, as illustrated in Figure 1A. Within the analysed cohort, 55 (82%) patients had a history of smoking, 32 being former smokers and 23 current smokers. Six individuals identified themselves as non-smokers, while for another six the smoking status was unavailable. This underscores an association between bladder cancer incidence and smoking habits, a well-established risk factor that triples the relative risk of bladder cancer compared to never smoking [50]. Approximately 43% of patients (29 in total) were diagnosed with non-invasive carcinoma (pTa), out of which 27 were treated with sparing surgery (TURBT) and 22 carcinomas that were assessed as low grade (i.e., having cancer cells that are well differentiated). For 19% of patients (13 in total), the invasive carcinoma was classified as pT1 and pT2, while for 34% (23 in total) stage pT3 and pT4 were observed. Nine individuals (13%) exhibited the presence of synchronous primary malignancies in both prostate and bladder cancer, a phenomenon that has been previously documented. The co-occurrence of prostate cancer in patients with bladder cancer has been reported to be as high as 70% [51]. Seven patients (10%) received preoperative intravesical treatment: three were treated with BCG immunotherapy and four with neoadjuvant chemotherapies (Table S1).

The collection protocol was designed in such a way that PT and PMs/DMs were collected and snap frozen up to 2 h from the organ resection; thus, they were initially only assessed macroscopically and presumed as normal or suspected to contain tumor cells. Subsequently, after freezing, all specimens underwent microscopic examination by a pathologist as part of the clinical routine to verify the actual tumor presence or absence (Material and Methods). During this stage, we noted that numerous samples that were supposed to be composed of exclusively histologically normal urothelium (PMs and DMs) contained cancer cells. On the other hand, we also noticed that several PT samples, which should contain tumor cells, consisted solely of normal urothelium. As summarized in Table 1 and Table S2, this reclassification affected 31% of the donors. In brief, 43 PMs and DMs from 21 donors were reclassified as containing cancer cells, while in six PTs from four donors no cancer cells were found. This underscores the critical importance of such verification, especially for PMs and DMs, which might affect the outcomes of possible correlations with patient survival and tumor relapse. Table 1 shows a trend for PMs having many more reclassifications than DMs. However, the difference has not shown statistical significance (not shown), likely due to the size of the cohort.

### 3.2. Detection of Autosomal Chromosomal Alterations (ACAs) in Histologically Normal Urothelium of Bladder Cancer Patients

Genotyping of the BLCA cohort for a total of 485 samples (Table 1) was performed using Illumina Infinium array and for the detection of ACAs such as gains, losses and CN-LOHs, we used the Mosaic Chromosomal Alterations (MoChA) software version 2022-05-18 [35]. In brief, haplotype phasing allows MoChA to detect allelic imbalances in the inherited 1:1 ratio of maternal and paternal chromosomal segments. We conducted quality control to eliminate samples that displayed signs of contamination or incomplete hybridization (see Methods). The hidden Markov-model-based approach of MoChA has a detection limit of approximately 1% of cellular fraction for ACAs. In 485 samples from all tissues, we identified 7987 ACAs including 2885 losses, 2554 gains and 2372 CN-LOHs, as well as 176 “Undetermined”, which accounted for ACAs with uncertain copy-number state (Table 2). Raw MoChA output annotated with germline/post-zygotic classification and manual curation notes is given in Table S3.

### 3.3. Distinction between Germline and Post-Zygotic ACAs in Non-Cancerous Samples

We concentrated the analysis on non-germline ACAs in histologically normal urothelium of the bladder. Generally speaking, the separation between inherited via germline and acquired post-zygotic (PZ) alterations might be problematic in studies using only one normal control tissue, especially if PZ-ACA occurs in a substantial number of cells. Here, we took advantage of our multi-tissue collection protocol to filter out germline ACAs. An ACA was categorized as germline if it was consistently detected in all non-cancerous PM and DM samples as well as in blood from the same subject. We also applied the single sample-based heuristic filtering from the MoChA pipeline for identification of germline variants. In 2385 ACAs from normal urothelium (Table 2; 1451 + 934), MoChA identified 62 (2%) ACAs as PZ-ACA, while our multi-tissue approach identified these as germline. On the other hand, MoChA marked 541 (19%) ACAs as germline, while our approach provided evidence that they were of PZ type. In summary, both approaches agreed on 2248 (79% of 2385) ACAs. Following this, we took the conservative approach and selected for further analysis only 934 PZ-ACAs, identified as such via both methods in the normal urothelium.

### 3.4. PZ-ACAs That Might Be Cancer Precursor Candidates (CPCs)

The main goal of this study was to identify the early aberrations present in histologically normal urothelium (i.e., PMs and DMs), under the assumption that these ACAs may have preconditioned the urothelium for BLCA development. The next step in our analysis was the visual inspection of 934 PZ-ACAs. This resulted in formulation of the following filtering step allowing to focus on the most apparent CPCs minimizing false positives. Of the 934 PZ-ACAs, we removed “Undetermined” calls, i.e., calls with cell fraction <5%, and calls with missing BAF or phasing evidence. Furthermore, we required good visual evidence from the LRR, BAF and phased BAF plots. The final step was a requirement to have at least 50% of CPC overlap with ACAs in at least one cancerous sample from the same donor, while having zero overlap between CPC and ACAs in blood of the same donor. The final step was a requirement to have at least 50% of CPC overlap with ACAs in at least one cancerous sample from the same donor, while having zero overlap between CPC and ACAs in blood of the same donor. In this way, we identified 480 manually curated CPCs in normal urothelium, summarized as “curated” in Table 2 and Table S4. We are aware that our filtering might be too conservative, leaving some false negatives, but this is in line with our goal of showing well-supported evidence for the existence of CPCs that may predispose to BLCA. Figures S1–S4 show the representative examples illustrating our logic for manual curation of CPCs. Additionally, we also tested for the possible influence of preoperative treatment on the mutational burden of normal urothelium and found no significant differences (details not shown). It should be stressed, however, that only seven patients received such treatment.

### 3.5. Recurrent Alterations and Cancer Driver Genes among Cancer Precursor Candidates (CPCs)

**Figure 2:**
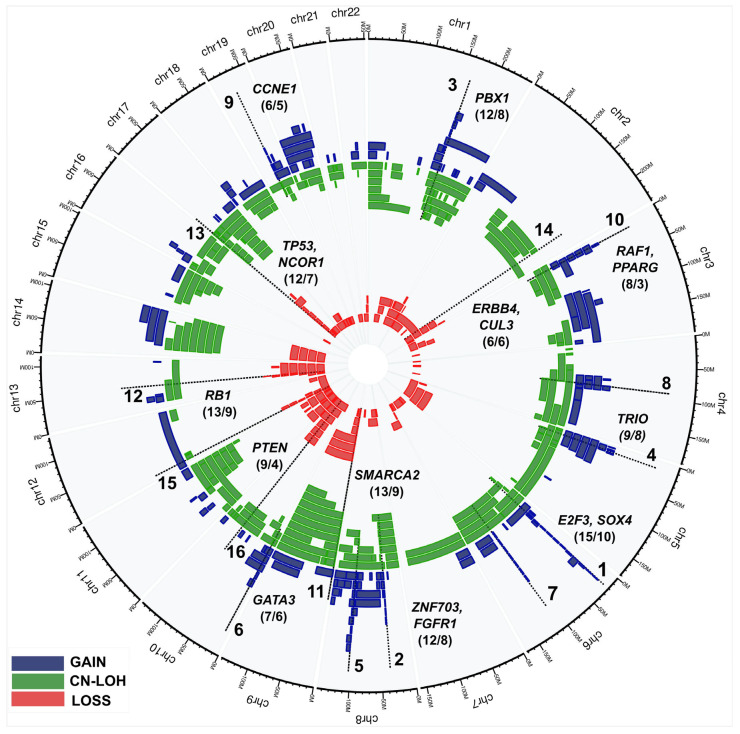
Genomic location and recurrence of cancer precursor candidates (CPCs) in samples of histologically normal urothelium. Analysis of gains (blue), losses (red) and CN-LOHs (green) among 480 CPCs in 64 NC samples of 30 patients yielded 16 hotspots of gain&CN-LOH or loss&CN-LOH type. Well-known cancer genes targeted by these hotspots are displayed as gene symbols, followed by the numbers in parentheses counting affected PM/DM samples and donors. For individual samples, CPCs shared by the same subject were counted several times. Table 3 and Table S5 show the hotspot’s GRCh37 coordinates, lengths and other genes previously implicated in bladder cancer. Abbreviations: CN-LOH: copy neutral loss of heterozygosity; hotspot: the shortest overlapping region of highest recurrence among patients and individual samples. Numbers before /-sign and after /-sign indicate numbers of patients and samples, respectively.

As mentioned above, our analysis was concentrated on PZ-ACAs present in 239 PMs and 75 DMs with normal histology. Overall, 45% of BLCA patients (30 out of 67) had at least one call in at least one sample of normal urothelium. This is considerably higher than previously reported for BLCA (18%) [30,31], and the corresponding number for sporadic breast cancer (38%) [43]. In 30 BLCA patients, 48% (64 out of 134 PMs/DMs) of samples had at least one alteration (Table S3). On the other hand, only 6 donors have 100% of PMs/DMs with at least one alteration. Most of the CPCs (386) in normal urothelium originate from 49 samples from cystectomy patients, while 94 CPCs come from 15 samples from TURBT donors. However, there was no bias for CPC counts per sample between cystectomy and TURBT patients. Our further analysis pinpointed 16 recurrent hotspots (shortest overlapping chromosomal regions) among 480 CPCs from 30 patients in normal urothelium (Table 2) shared across multiple donors and samples. Figure 2 and Table 3 show the whole genome landscape of CPCs along with identified hotspots. Allelic substitutions produced by CN-LOH can either duplicate or delete the alleles related to cancer development. Therefore, we defined hotspots by combining CN-LOH with gains (Gain&CN-LOH) as well as CN-LOH with deletions (Loss&CN-LOH). The underlying premise was that oncogenes within hotspots affected by Gain&CN-LOH and tumor suppressor genes affected by Loss&CN-LOH are the primary candidates for involvement in BLCA initiation.

Candidate genes within each hotspot were chosen based on their oncogenic potential. The assessment of oncogenic effects was performed using OncoKB, a manually curated knowledge base for cancer genes from Memorial Sloan Kettering Cancer Center [52] and Cancer Genome Atlas Research Network [33]. We identified 10 gain&CN-LOH hotspots distributed across eight autosomes, with varying lengths, spanning from 0.8 to 12.2 Mb. These were detected in 4–9 individual donors, and 6–15 samples (Figure 2 and Table 3). We further delineated six Loss&CN-LOH hotspots, characterized by substantially broader alterations, spanning 5.1–56.2 Mb across six autosomes, in 4–9 donors and 6–13 samples. As could be expected, these hotspots consistently target many well-established cancer driver genes.

Many of the identified Gain&CN-LOH hotspots target genes that are commonly amplified in BLCA. These genes include *E2F3*, *SOX4*, *FGFR1*, *ZNF703*, *GATA3*, *CUL3*, *CCNE1* and *PPARG* [33]. Additionally, some genes, while not typically linked to BLCA, were considered as candidate genes in our analysis based on supporting evidence from the cancer literature. These include *PBX1*, *TRIO* and *RAF1.* Similarly, the majority of the Loss&CN-LOH hotspots encompass tumor suppressor genes already known to be deleted in BLCA. This group includes genes such as *SMARCA2*, *RB1*, *TP53*, *NCOR1* and *PTEN*. Furthermore, some genes, while having a less established role in BLCA compared to other cancers, were also implicated in the Loss&CN-LOH hotspots, such as *ERRB4*.

The most prominent among the recurrent alteration is hotspot #1. This 6p22.3 region is increased in copy number in 10–20% of BLCA tumors [53,54] and is defined based on alterations in 10 donors and 15 samples and encompasses well-known genes involved in BLCA such as *E2F3* and *SOX4*. The additional 15 hotspots are also involved in aberrations detected in BLCA tumors, as shown in detail in Figure 2 and Table 3 and Table S4. In the case of hotspot #7, we have not been able to identify any clear candidate genes. A cross-hotspot analysis of functional pathways of the most obvious cancer driver genes further reinforces the relevance of these aberrations in cancer development. For instance, six hotspots (numbers 1, 4, 9, 12, 13 and 15) encompass at least six well-studied genes important for cell-cycle regulation (*E2F3*, *CCNE1*, *TRIO*, *CDKN1B*, *RB1* and *TP53*) (Table 3 and Table S5). Hotspots 2 and 14 contain two well-known receptor tyrosine kinases implicated in development of multiple cancers (*FGFR1* and *ERBB4*), but they are very infrequently mentioned in the BLCA literature. Moreover, hotspots 10 and 16 contain genes involved in PI3K-AKT, MAPK and WNT intracellular signalling pathways (*PTEN*, *RAF1* and *BMPR1* genes). In conclusion, the above-described analysis of normal urothelium from BLCA patients provides a proof of principle that the approach we used is viable in characterization of the early genetic alterations that may predispose to cancer development.

### 3.6. Loss of Chromosome Y (LOY) in Multiple Tissues from Male Bladder Cancer Patients

#### 3.6.1. Concordance of LOY Estimation between MoChA and ddPCR

**Figure 3:**
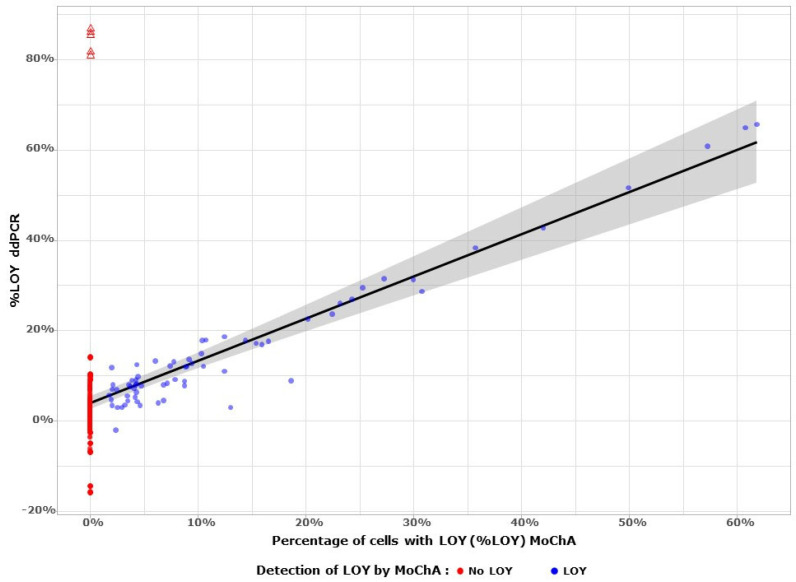
Comparison of performance for LOY estimation between MoChA and ddPCR. The external cohort of blood samples (*n* = 273) was used to compare LOY estimation via MoChA (%LOY on the X axis) and ddPCR (%LOY on the Y axis). When MoChA detects LOY (blue circles), %LOY agrees well with ddPCR values up to values < 65%. The samples where MoChA does not detect any LOY are represented by red color. Five red triangles benefit from ddPCR verification as they have very high %LOY and these samples do have enough heterogeneous probes within PAR1 for MoChA to reliably compute BAF deviation. The grey area represents the standard error of the linear regression model (%LOY ddPCR) = α + β (%LOY MoChA) where α = 0.02 (95% CI [0.013, 0.036]) and β = 0.99 (95% CI [0.93, 1.05], *p* < 1 × 10^–3^ ***). The Pearson’s correlation coefficient for 67 samples where MoChA detects LOY was r = 0.97 (95% CI [0.96, 0.98], *p* < 1 × 10^−3^ ***, R^2^ = 0.94). This shows that MoChA is a reliable tool for estimating LOY in blood samples, up to values of about 65%.

**Figure 4:**
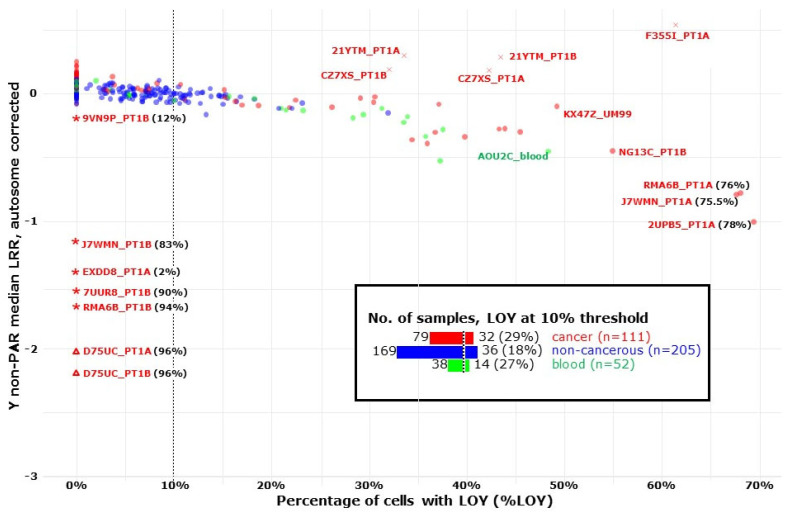
Mosaic loss of chromosome Y (LOY) in samples from bladder cancer cohort. This figure shows the median log R ratio (LRR) of the non-PAR region of chromosome Y, adjusted by median LRR of autosomes, and the percentage of cells with LOY (%LOY), estimated via MoChA using B-allele frequency (BAF) deviation of PAR1 region of chromosome Y, for 52 male patients of the BLCA cohort. Blood samples (*n* = 52, green), cancer samples (*n* = 111, red) and non-cancerous samples from bladder mucosa (*n* = 205, blue) are shown. Sample KX47Z_UM99 has been reclassified as a tumor sample after histopathological analysis. Five samples (red crosses) with either gain of chromosome Y (GOY) or XXY genotype are upper outliers and all these are tumor samples. The inset summarizes the number and percent of samples at the conservative threshold of LOY > 10% for three types of samples. ddPCR was used to validate %LOY (shown as percentages after sample label) for outliers without enough heterozygous probes, where BAF deviation is not reliably measured. Out of 7 left-bottom outliers, MoChA was able to detect LOY for *n* = 2 samples (triangles), but underestimated its cellular fraction, LOY < 10% was correctly detected by MoChA for EXDD8_PT1A sample, and LOY > 10% was missed by MoChA for *n* = 5 (asterisks) samples, which illustrate the need to complement MoChA’s output with other methods. Three samples in the rightmost part of the LOY cloud, deviating from the linear trend, are where MoChA underestimates %LOY and these were also validated with ddPCR. Abbreviations: PAR, pseudoautosomal region of chromosome Y; PAR1, pseudoautosomal region 1.

In addition to ACAs, MoChA software is also able to detect mosaic loss of chromosome Y (LOY) [35]. This software uses haplotype phasing information to detect chromosomal alterations and for the purpose of LOY-analysis uses allelic imbalances at heterozygous sites within pseudoautosomal region 1 (PAR1) of the sex chromosomes. The earlier LOY analysis procedures were based on LRR deviation of SNP array probes of the male-specific region of the Y chromosome (MSY) [6] and droplet digital PCR (ddPCR) [25]. The above methods vary in sensitivity due to the different algorithms, leading occasionally to diverging results [19,23,55]. In this LOY analysis of the BLCA cohort, the first step was an evaluation of the precision of MoChA-based LOY scoring, comparing it with ddPCR. We used for this purpose an external anonymized cohort of 273 blood samples from Uppsala Academic Hospital, in which LOY measurements were previously obtained with ddPCR and Illumina SNP-array [25]. When MoChA detects LOY (Figure 3, blue circles), %LOY agrees very well with ddPCR (Pearson correlation coefficient r = 0.97, 95% CI [0.96, 0.98], *n* = 67, *p* < 1 × 10^−3^ ***). However, when the cellular fraction of %LOY is high, MoChA underestimates LOY because heterozygous single-nucleotide polymorphisms are “dropping out” of the data for large BAF deviations [56], as indicated by the five samples shown as red triangles in Figure 3. Therefore, while MoChA is a reliable tool for estimating %LOY in samples up to values of 65%, the verification using another method is recommended for samples where high %LOY values are suggested by low median LRRs of non-PAR regions of chromosome Y (Figure 4, triangles and asterisks).

#### 3.6.2. The Analysis of LOY Status in the BLCA Cohort Using Three Types of Tissues

We next used MoChA to score LOY in 376 samples derived from 52 male BLCA patients. Among these, 52 samples were from blood, 111 were PTs, and 205 were non-cancerous PM and DM samples. Five PTs (Figure 4, red crosses) showing either mosaic gain of chromosome Y (GOY) or XXY genotype, were identified as upper outliers by their median LRRs of non-PAR regions of chromosome Y and were not included in further analysis. The remaining samples were classified as LOY < 10%, when LOY was not detected or its cellular fraction was less than 10%. With this threshold, 18% of non-cancerous samples showed LOY > 10%, which is significantly lower than 29% of PTs (*p* = 0.037, Fisher’s exact test), and also lower than 27% of blood samples (inset in Figure 4).

The latter comparison was a trend, due to the low number of blood samples (*p* < 0.1, Fisher’s exact test). However, in samples with very high LOY, the cellular fraction of LOY cannot be calculated reliably via MoChA, due to the aforementioned “dropping out” [56] of heterozygous probes (7 outliers on the Y-axis, triangles and asterisks in Figure 4). DNA from these samples was re-analyzed using the ddPCR-based LOY assay and, indeed, high levels of LOY were confirmed (Figure 4, %LOY values in parentheses). In addition, we validated with ddPCR three additional samples (RMA6B_PT1A, J7WMN_PT1A and 2UPB5_PT1A) that were located at the right-end of the cloud of MoChA-LOY measurements in Figure 4. The latter results further confirm that MoChA underestimates %LOY in samples with >65% cellular fraction of LOY, with a relatively sharp cut-off. Generally speaking, our LOY results show a wide range of %LOY values in the BLCA cohort, from zero to 96%, with the highest values detected in tumor samples.

#### 3.6.3. LOY Co-Occurrence between Blood, Non-Cancerous and PT Samples

**Figure 5:**
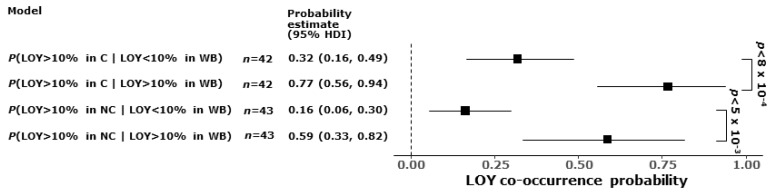
Conditional probabilities of LOY co-occurrence between cancerous (C), non-cancerous (NC) and whole blood (WB) samples. Probability of detecting LOY > 10% in at least one C or NC sample is significantly higher if WB sample of this donor has LOY > 10%. Points show medians, horizontal bars show 95% HDI from Bayesian regression, controlling for age, age^2^ and smoking history confounders, removing donors where data were missing. *p*-values are Bonferroni-adjusted. There are 42 donors with non-missing LOY, age and smoking data in both C and WB tissues, and 43 donors with non-missing LOY, age and smoking data in both NC and WB tissues.

In order to determine the statistical probability of LOY co-occurrence in different tissues, we evaluated this probability by comparing the level of LOY in samples with a 10% threshold (LOY > 10%, Figure 4 and Figure 5). We excluded from this analysis donors with atypical histology, multifocal cancer and PT samples that were reclassified as non-cancerous. The conditional probability of LOY co-occurrence was tested between cancerous (C), non-cancerous (NC) and whole blood (WB) samples from 47 male BLCA patients, adjusting for age, age^2^ and smoking history (Figure 5).

For example, P(LOY > 10% in C | LOY < 10% in WB) stands for the probability of detecting at least one C sample with LOY > 10% for a donor with LOY < 10% in WB. The analysis showed that the co-occurrence of P(LOY > 10% in C | LOY > 10% in WB) had a median probability of 0.77 (95% HDI: 0.56 to 0.94), which was significantly higher (median difference: 0.44, 95% HDI: 0.19 to 0.67, Bonferroni adjusted *p* < 8 × 10^−4^) than the co-occurrence of P(LOY > 10% in C | LOY < 10% in WB) with a median probability of 0.32 (95% HDI: 0.16 to 0.49) (Figure 5). Additionally, the co-occurrence of P(LOY > 10% in NC | LOY > 10% in WB) had a median probability of 0.59 (95% HDI: 0.33 to 0.82), which was significantly higher (median difference: 0.42, 95% HDI: 0.15 to 0.67, Bonferroni adjusted *p* < 5 × 10^−3^) than the co-occurrence of P(LOY > 10% in NC | LOY < 10% in WB) with a median probability of 0.16 (95% HDI: 0.06 to 0.3). Thus, LOY in different tissues of the same donor showed significant co-occurrence beyond what could be attributed just to age and smoking history. When histologically normal urothelium and PT in BLCA patients are considered, this might be due to the ongoing cancerization process of urothelial cells.

## 4. Discussion

The basic premise of our study is the concept of aberrant clonal expansions of cells in morphologically normal tissues [45] and this is best studied in the hematopoietic system. It is termed clonal hematopoiesis [57,58], where the most prevalent alteration is LOY, a mutation also linked to various diseases in men [6,16,20,21,22,23,24,25,26]. These aberrant clonal expansions have also been described in solid tissues, often in connection with cancer, and are commonly referred to as field cancerization [46,47,48,49,59,60,61]. The novelty of our approach lies in the experimental design based on profiling the landscape of chromosomal alterations across several tissues, including multiple non-cancerous margin samples (2–8 per patient) located at different distances from the tumor (PMs and DMs). Furthermore, our current BLCA data are well aligned with our previous studies of histologically normal margins of breast tissue from breast cancer patients [42,43,44,45].

We combined this multi-sample- and multi-tissue-per-patient approach with an advantage of a sensitive algorithm for detection of chromosomal aberrations present at low cellular fraction (MoChA) and comparison across normal and tumor samples. This allows uncovering the early alterations that might predispose to the development of BLCA. The process of accumulation of mutations might create a pro-tumorigenic environment, which can drive the growth and spread of cells in the bladder and ultimately be accompanied by the loss of normal tissue architecture typical for cancer [1]. Yet another advantage of our approach is allowing us to reliably distinguish between the inherited via germline and true acquired post-zygotic (PZ) alterations. Generally speaking, the normal urothelium cells can acquire a considerable number of genetic aberrations before their abnormal histology will be seen under the microscope, which is reminiscent of results previously shown for breast cancer patients [42,43,44,45].

Our analysis reveals widespread CPCs of different sizes, which may affect a considerable number of tumor suppressors or oncogenes. These mutations may promote tumor formation either by direct initiation of malignant transformation or by creating a tumor-permissive environment within the tissue. The identification of 16 CPC hotspots in proximal margins (PMs) and distal margins (DMs) from 30 BLCA patients was of particular interest. Many of these hotspots resemble typical aberrations described in previous studies of bladder tumors. For instance, the gain&CN-LOH hotspot #1, affecting *E2F3* and *SOX4*, is the most frequent aberration in bladder tumors [62,63]. Furthermore, the gains on 8p11 encompassing *FGFR1* and *ZNF703* is the second most common hotspot in our analysis (#2), which agrees with previous tumor-related literature [64]. The third one is loss&CN-LOH on 9p (hotspot #11) that may target several tumor suppressors, among them *SMARCA2*, *NFIB* and *PTPRD* (Table 3 and Table S4). On the other hand, there were also differences in the alteration landscape between our results and previous tumor-based studies. The gain&CNLOH encompassing the *FGFR1* gene is our prominent result, albeit this gene has not been frequently described previously in the BLCA literature. Another distinctive example in our study is the common loss&CN-LOH of *ERBB4* and several other tumor suppressor candidates (hotspot #14), while tumor studies point to more prevalent involvement of *ERBB2* and *ERBB3.* Finally, loss&CN-LOH of *CDKN1B* (hotspot #15) is frequent in our results, but tumor-related analyses are pointing to another member of cyclin-dependent kinases, such as *CDKN2A*.

Moreover, our study should also be discussed in the context of male bias for BLCA, with men being four times more likely than women to develop the disease [1,3,4,5]. The underlying cause of this male predominance is not yet fully understood. However, there is increasing evidence supporting the importance of LOY as a genetic factor predisposing to male cancers [6,7,17,65]. Our analyses revealed that at least 27% of blood samples and at least 29% of PTs had LOY above 10%, but only 18% of non-cancerous samples showed LOY in more than 10% of cells. There might be several explanations for this difference. LOY occurs as a consequence of an error in mitosis and is therefore most frequent in tissues with high daily turnover. Normal urothelium, recognized as one of the slowest dividing epithelia in the human body, do not undergo as many cell divisions as tumor cells and circulating leukocytes. Furthermore, it has been shown that tumors have a higher number of infiltrating leukocytes than healthy tissue [66,67]. The above explanation might be true under the assumption that the observed LOY in tumors is mainly due to infiltrating leukocytes affected by LOY. Our independent analyses identified T cells, and among them regulatory T cells, to be frequently affected by LOY in the tumor microenvironment (TME) [68]. An alternative and not mutually exclusive explanation might be that tumors propagate cells with LOY, as it provides a proliferative advantage to cell clones with this aneuploidy [67].

While our study provides valuable insights into understanding the pathogenesis of BLCA, we acknowledge that it has some limitations. Although we present the genetic architecture of CPCs in normal urothelium from patients with BLCA, 55% of patients are free of large post-zygotic alterations in normal urothelium detectable via MoChA and this category should be investigated for the presence of other types of mutations, such as point mutations and epigenetic modifications of DNA/chromatin. Indeed, similar studies utilizing targeted sequencing and more sensitive duplex sequencing, reveal frequent mutations in cancer-driver genes, which could be primers of tumorigenesis [44,69,70]. Moreover, the above-mentioned changes are likely going to affect the global portrait of transcriptome and this should also be considered in future studies, as it may lead to description of gene expression signature related to a higher risk of BLCA. Another limitation is that 80% of CPCs are detected in cystectomy patients, which is due to the comprehensive nature of sample collection for cystectomy patients, involving the removal of the entire bladder, providing many more samples per patient. However, there was no bias for CPC counts per sample between cystectomy and TURBT patients. Eventually, results of extended analysis similar to those reported here, have a potential for improvement of early diagnostics of BLCA, via detection of mutations in the urine of patients at risk, such as heavy smokers or subjects exposed to various occupational challenges. Thus, future studies on larger cohorts of bladder cancer patients will be necessary to better understand the picture of PZ-ACAs and LOY in BLCA. Our results provide insights into mechanisms of BLCA development and may have important implications for the designing of new strategies for the prevention and treatment of BLCA.

## 5. Conclusions

We developed a methodological pipeline for sensitive identification and comparisons of post-zygotic chromosomal alterations across different tissues from the same BLCA patient. This method is based on the MoChA algorithm allowing the detection of autosomal deletions, gains and CN-LOH as well as LOY in normal urothelium, which are present at low cellular fraction. This combination of multiple normal samples and a sensitive detection method for chromosomal alterations permits the identification of the early alterations present in urothelium from BLCA patients. Based on this approach, we reported 16 hotspots of recurring changes that target many well-established cancer driver genes derived from analysis of BLCA tumors. However, we also reported gene regions not frequently detected in previous analyses.

We also studied LOY and confirmed its connection to BLCA development. Although LOY has recently been shown as the causative event in the evolution of BLCA using the mouse model, our results show that LOY is not uniformly distributed in normal urothelium, tumor and peripheral leukocytes. Using a conservative threshold of at least 10% of cells with LOY, samples of tumors, blood and normal urothelium showed 29%, 27% and 18% of LOY, respectively. This may suggest that LOY in blood and normal urothelium is due to independent mutational events in different cell lineages, meaning that LOY occurs several times during a lifetime of the same male subject. This warrants future research into the role of LOY in BLCA and its potential for being a marker for higher risk or worse prognosis of BLCA in males.

## Figures and Tables

**Table 1:** Sample counts and histopathological reclassification of solid tissues in the BLCA cohort.

Tissue Collection Site	Reclassification Summary		Total
	Cancerous (C)	Non-Cancerous (NC)	
Primary tumors (PTs)	102 (94.4%)	**6 (5.6%)**	108
Proximal margins (PMs)	**37 (15.5%)**	202 (84.5%)	239
Distal margins (DMs)	**6 (8%)**	69 (92%)	75
Whole blood (WB)	-	-	63
Total			485

Solid tissues containing cancer cells were classified as cancerous (C) and samples with normal histology were classified as non-cancerous (NC). Samples marked in bold were reclassified.

**Table 2:** The output of Mosaic Chromosomal Alteration (MoChA) software.

ACA Classification	Sample Type	CN-LOH	Gain	Loss	Undetermined	Total Alterations
Germline	WB	1	112	212	1	326
	NC	16	546	851	38	1451
Post-zygotic	WB	0	2	0	0	2
	NC	492	230	157	55	934
	(NC curated *)	(200 *)	(167 *)	(113 *)	(- *)	(480 *)
In cancerous samples	C	1863	1664	1665	82	5274
**Total**		2372	2554	2885	176	7987

Abbreviations: ACA, autosomal chromosomal alteration; CN-LOH, copy neutral loss of heterozygosity; WB, whole blood; NC, non-cancerous solid tissue, i.e., proximal margin and distal margin samples; C, cancerous solid tissue; * indicates ACAs after manual curation.

**Table 3:** Cancer precursor candidates defined by recurrent alteration hotspots and cancer driver genes located therein.

ACA Hotspot *		Genomic Coordinate	Cytoband	Size (Mb) *	No. Samples	No. Donors	Candidate Gene(s) **
Gain&CN-LOH	1	6:20556906-22194664	6p22.3	1.6	15	10	**E2F3**, **SOX4**
	2	8:37730457-38480529	8p11.23	0.8	12	8	**ZNF703**, **FGFR1**
	3	1:160899482-161794336	1q23.3	0.9	12	8	**PBX1**, NCSTN, DDR2, NUF2
	4	5:7838086-15742599	5p15.31-p15.1	7.9	9	8	**TRIO**, CTNND2, CCT5, DNAH5, SEMA5A
	5	8:101206392-103578988	8q22.3	2.4	13	6	UBR5
	6	10:3896352-13730665	10p15.1-10p13	9.8	7	6	**GATA3**
	7	6:105973888-108499945	6q21	2.5	11	5	
	8	4:71218055-83369287	4q13.3-4q21.23	12.2	7	5	AFF1
	9	19:29712442-30577229	19q12	0.9	6	5	**CCNE1**
	10	3:11432237-15082196	3p25.3-3p35.1	3.6	8	3	**RAF1**, **PPARG**
Loss&CN-LOH	11	9:1-21413703	9p24.3-9p21.3	21.4	13	9	**SMARCA2**, NFIB, PTPRD
	12	13:44640316-51689586	13q14.12-13q14.3	7.1	13	9	**RB1**, RNASEH2B, SETDB2, INTS6
	13	17:3649573-17235537	17p13.2-17p11.2	13.6	12	7	**TP53**, **NCOR1**, MAP2K4, PRPF8, GPS2, ALOX12B, FLCN
	14	2:205942561-243199373	2q33.3-2q37.3	36.3	6	6	**ERBB4**, CUL3, PTMA, SP140, HDAC4, BARD1, INHA
	15	12:12028158-17130524	12p12.2-12p12.3	5.1	7	5	CDKN1B, ETV6
	16	10:57545821-113719764	10q21.1-10q25.3	56.2	9	4	**PTEN**, ARID5B, BMPR1A, CCDC6, SMC3, FAS, TET1, SUFU

* Hotspot is a shortest overlapping chromosomal region shared across multiple donors and samples. ** Genes in bold text are also shown in Figure 2 and represent well-known cancer driver genes.

## Data Availability

De-identified individual data might be made available following publication by reasonable request to the corresponding authors.

## Supplementary Materials

The following supporting information can be downloaded at: https://www.mdpi.com/article/10.3390/cancers16050961/s1, Table S1: Patient summary; Table S2: Summary of the reclassification outcomes for solid tissue samples, based on results from histopathological examination;; Table S3: Raw MoChA output with manual curation and annotation; Table S4: Per patient distribution of cancer precursor candidates (CPCs) in histologically normal urothelium; Table S5: The functional pathways for the cancer precursor candidate (CPC) genes located in genetic hotspots; Figures S1–S4: Representative examples of manual curation of CPCs.

## References

[1] Dyrskjøt L., Hansel D.E., Efstathiou J.A., Knowles M.A., Galsky M.D., Teoh J., Theodorescu D. (2023). Bladder cancer. Nat. Rev. Dis. Primers.

[2] Facchini G., Cavaliere C., Romis L., Mordente S., Facchini S., Iovane G., Capasso M., D’Errico D., Liguori C., Formato R. (2020). Advanced/metastatic bladder cancer: Current status and future directions. Eur. Rev. Med. Pharmacol. Sci..

[3] Sung H., Ferlay J., Siegel R.L., Laversanne M., Soerjomataram I., Jemal A., Bray F. (2021). Global Cancer Statistics 2020: GLOBOCAN Estimates of Incidence and Mortality Worldwide for 36 Cancers in 185 Countries. CA Cancer J. Clin..

[4] Safiri S., Kolahi A.A., Naghavi M. (2021). Global, regional and national burden of bladder cancer and its attributable risk factors in 204 countries and territories, 1990–2019: A systematic analysis for the Global Burden of Disease study 2019. BMJ Glob. Health.

[5] Doshi B., Athans S.R., Woloszynska A. (2023). Biological differences underlying sex and gender disparities in bladder cancer: Current synopsis and future directions. Oncogenesis.

[6] Forsberg L.A., Rasi C., Malmqvist N., Davies H., Pasupulati S., Pakalapati G., Sandgren J., de Stahl T.D., Zaghlool A., Giedraitis V. (2014). Mosaic loss of chromosome Y in peripheral blood is associated with shorter survival and higher risk of cancer. Nat. Genet..

[7] Abdel-Hafiz H.A., Schafer J.M., Chen X., Xiao T., Gauntner T.D., Li Z., Theodorescu D. (2023). Y chromosome loss in cancer drives growth by evasion of adaptive immunity. Nature.

[8] Dumanski J.P., Rasi C., Lonn M., Davies H., Ingelsson M., Giedraitis V., Lannfelt L., Magnusson P.K., Lindgren C.M., Morris A.P. (2015). Smoking is associated with mosaic loss of chromosome Y. Science.

[9] Panani A.D., Roussos C. (2006). Sex chromosome abnormalities in bladder cancer: Y polysomies are linked to PT1-grade III transitional cell carcinoma. Anticancer Res..

[10] Fadl-Elmula I., Gorunova L., Mandahl N., Elfving P., Lundgren R., Mitelman F., Heim S. (2000). Karyotypic characterization of urinary bladder transitional cell carcinomas. Genes Chromosomes Cancer.

[11] Sauter G., Moch H., Mihatsch M.J., Gasser T.C. (1998). Molecular cytogenetics of bladder cancer progression. Eur. Urol..

[12] Smeets W., Pauwels R., Laarakkers L., Debruyne F., Geraedts J. (1987). Chromosomal analysis of bladder cancer. III. Nonrandom alterations. Cancer Genet. Cytogenet..

[13] Sauter G., Moch H., Wagner U., Novotna H., Gasser T.C., Mattarelli G., Mihatsch M.J., Waldman F.M. (1995). Y chromosome loss detected by FISH in bladder cancer. Cancer Genet. Cytogenet..

[14] Neuhaus M., Wagner U., Schmid U., Ackermann D., Zellweger T., Maurer R., Alund G., Knönagel H., Rist M., Moch H. (1999). Polysomies but not Y chromosome losses have prognostic significance in pTa/pT1 urinary bladder cancer. Hum. Pathol..

[15] Powell I., Tyrkus M., Kleer E. (1990). Apparent correlation of sex chromosome loss and disease course in urothelial cancer. Cancer Genet. Cytogenet..

[16] Dumanski J.P., Lambert J.C., Rasi C., Giedraitis V., Davies H., Grenier-Boley B., Lindgren C.M., Campion D., Dufouil C., European Alzheimer’s Disease Initiative Investigators (2016). Mosaic Loss of Chromosome Y in Blood Is Associated with Alzheimer Disease. Am. J. Hum. Genet..

[17] Thompson D., Genovese G., Halvardson J., Ulirsch J., Wright D., Terao C., Davidsson O., Day F., Sulem P., Jiang Y. (2019). Genetic predisposition to mosaic Y chromosome loss in blood. Nature.

[18] Dumanski J., Halvardson J., Davies H., Rychlicka-Buniowska E., Mattisson J., Torabi Moghadam B., Nagy N., Węglarczyk K., Bukowska-Strakova K., Danielsson M. (2021). Immune cells lacking Y chromosome show dysregulation of autosomal gene expression. Cell. Mol. Life Sci..

[19] Forsberg L., Halvardson J., Rychlicka E., Danielsson M., Torabi Moghadam B., Mattisson J., Rasi C., Davies H., Lind L., Giedraitis V. (2019). Mosaic loss of chromosome Y (LOY) in leukocytes matters. Nat. Genet..

[20] Loftfield E., Zhou W., Graubard B.I., Yeager M., Chanock S.J., Freedman N.D., Machiela M.J. (2018). Predictors of mosaic chromosome Y loss and associations with mortality in the UK Biobank. Sci. Rep..

[21] Haitjema S., Kofink D., van Setten J., van der Laan S., Schoneveld A., Eales J., Tomaszewski M., de Jager S., Pasterkamp G., Asselbergs F. (2017). Loss of Y Chromosome in Blood Is Associated with Major Cardiovascular Events during Follow-up in Men after Carotid Endarterectomy. Circ. Cardiovasc. Genet..

[22] Sano S., Horitani K., Ogawa H., Halvardson J., Chavkin N.W., Wang Y., Sano M., Mattisson J., Hata A., Danielsson M. (2022). Hematopoietic loss of Y chromosome leads to cardiac fibrosis and heart failure mortality. Science.

[23] Vermeulen M.C., Pearse R., Young-Pearse T., Mostafavi S. (2022). Mosaic loss of Chromosome Y in aged human microglia. Genome Res..

[24] Dorvall M., Pedersen A., Dumanski J., Söderholm M., Lindgren A., Stanne T., Jern C. (2023). Mosaic loss of chromosome Y is associated with functional outcome after ischemic stroke. Stroke.

[25] Bruhn-Olszewska B., Davies H., Sarkisyan D., Juhas U., Rychlicka-Buniowska E., Wójcik M., Horbacz M., Jąkalski M., Olszewski P., Westholm J.O. (2022). Loss of Y in leukocytes as a risk factor for critical COVID-19 in men. Genome Med..

[26] Loftfield E., Zhou W., Yeager M., Chanock S.J., Freedman N.D., Machiela M.J. (2019). Mosaic Y Loss Is Moderately Associated with Solid Tumor Risk. Cancer Res..

[27] Martin B.F. (1972). Cell replacement and differentiation in transitional epithelium: A histological and autoradiographic study of the guinea-pig bladder and ureter. J. Anat..

[28] Hicks R.M. (1975). The mammalian urinary bladder: An accommodating organ. Biol. Rev. Camb. Philos. Soc..

[29] Wang C., Ross W.T., Mysorekar I.U. (2017). Urothelial generation and regeneration in development, injury, and cancer. Dev. Dyn..

[30] Lawson A.R.J., Abascal F., Coorens T.H.H., Hooks Y., O’Neill L., Latimer C., Raine K., Sanders M.A., Warren A.Y., Mahbubani K.T.A. (2020). Extensive heterogeneity in somatic mutation and selection in the human bladder. Science.

[31] Jakubek Y.A., Chang K., Sivakumar S., Yu Y., Giordano M.R., Fowler J., Huff C.D., Kadara H., Vilar E., Scheet P. (2020). Large-scale analysis of acquired chromosomal alterations in non-tumor samples from patients with cancer. Nat. Biotechnol..

[32] Czerniak B., Dinney C., McConkey D. (2016). Origins of Bladder Cancer. Annu. Rev. Pathol..

[33] Network C.G.A.R. (2014). Comprehensive molecular characterization of urothelial bladder carcinoma. Nature.

[34] Filipowicz N., Drężek K., Horbacz M., Wojdak A., Szymanowski J., Rychlicka-Buniowska E., Juhas U., Duzowska K., Nowikiewicz T., Stańkowska W. (2022). Comprehensive cancer-oriented biobanking resource of human samples for studies of post-zygotic genetic variation involved in cancer predisposition. PLoS ONE.

[35] Loh P.R., Genovese G., McCarroll S.A. (2020). Monogenic and polygenic inheritance become instruments for clonal selection. Nature.

[36] Stan Development Team RStan: The R Interface to Stan. R Package Version 2.21.2. http://mc-stan.org/.

[37] R Core Team (2010). R: A Language and Environment for Statistical Computing.

[38] Bürkner P.-C. (2017). brms: An R Package for Bayesian Multilevel Models Using Stan. J. Stat. Softw..

[39] McElreath R. (2020). Statistical Rethinking. A Bayesian Course with Examples in R and STAN.

[40] Gelman A. Prior Choice Recommendations. In Stan-Dev/Stan. Ed. GitHub. https://github.com/stan-dev/stan/wiki/Prior-Choice-Recommendations.

[41] Lenth R.V. emmeans: Estimated Marginal Means, aka Least-Squares Means. R Package Version 1.6.3. https://CRAN.R-project.org/package=emmeans.

[42] Ronowicz A., Janaszak-Jasiecka A., Skokowski J., Madanecki P., Bartoszewski R., Balut M., Seroczynska B., Kochan K., Bogdan A., Butkus M. (2015). Concurrent DNA Copy-Number Alterations and Mutations in Genes Related to Maintenance of Genome Stability in Uninvolved Mammary Glandular Tissue from Breast Cancer Patients. Hum. Mutat..

[43] Forsberg L.A., Rasi C., Pekar G., Davies H., Piotrowski A., Absher D., Razzaghian H.R., Ambicka A., Halaszka K., Przewoznik M. (2015). Signatures of post-zygotic structural genetic aberrations in the cells of histologically normal breast tissue that can predispose to sporadic breast cancer. Genome Res..

[44] Kostecka A., Nowikiewicz T., Olszewski P., Koczkowska M., Horbacz M., Heinzl M., Andreou M., Salazar R., Mair T., Madanecki P. (2022). High prevalence of somatic PIK3CA and TP53 pathogenic variants in the normal mammary gland tissue of sporadic breast cancer patients revealed by duplex sequencing. NPJ Breast Cancer.

[45] Forsberg L.A., Gisselsson D., Dumanski J.P. (2017). Mosaicism in health and disease—Clones picking up speed. Nat. Rev. Genet..

[46] Slaughter D.P., Southwick H.W., Smejkal W. (1953). “Field cancerization” in oral stratified squamous epithelium. Clinical implications of multicentric origin. Cancer.

[47] Lochhead P., Chan A., Nishihara R., Fuchs C., Beck A., Giovannucci E., Ogino S. (2015). Etiologic field effect: Reappraisal of the field effect concept in cancer predisposition and progression. Mod. Pathol..

[48] Curtius K., Wright N.A., Graham T.A. (2018). An evolutionary perspective on field cancerization. Nat. Rev..

[49] Höglund M. (2007). On the origin of syn- and metachronous urothelial carcinomas. Eur. Urol..

[50] Freedman N.D., Silverman D.T., Hollenbeck A.R., Schatzkin A., Abnet C.C. (2011). Association between smoking and risk of bladder cancer among men and women. JAMA.

[51] Kinoshita Y., Singh A., Rovito P.M., Wang C.Y., Haas G.P. (2004). Double primary cancers of the prostate and bladder: A literature review. Clin. Prostate Cancer.

[52] Chakravarty D., Gao J., Phillips S.M., Kundra R., Zhang H., Wang J., Rudolph J.E., Yaeger R., Soumerai T., Nissan M.H. (2017). OncoKB: A Precision Oncology Knowledge Base. JCO Precis. Oncol..

[53] Shen H., Morrison C.D., Zhang J., Underwood W., Yang N., Frangou C., Eng K., Head K., Bollag R.J., Kavuri S.K. (2013). 6p22.3 amplification as a biomarker and potential therapeutic target of advanced stage bladder cancer. Oncotarget.

[54] Wu Q., Hoffmann M.J., Hartmann F.H., Schulz W.A. (2005). Amplification and overexpression of the ID4 gene at 6p22.3 in bladder cancer. Mol. Cancer.

[55] Zhou W., Machiela M.J., Freedman N.D., Rothman N., Malats N., Dagnall C., Caporaso N., Teras L.T., Gaudet M.M., Gapstur S.M. (2019). Reply to ‘Mosaic loss of chromosome Y in leukocytes matters’. Nat. Genet..

[56] Watson C.J., Blundell J.R. (2023). Mutation rates and fitness consequences of mosaic chromosomal alterations in blood. Nat. Genet..

[57] Jaiswal S., Ebert B.L. (2019). Clonal hematopoiesis in human aging and disease. Science.

[58] Evans M.A., Walsh K. (2023). Clonal hematopoiesis, somatic mosaicism, and age-associated disease. Physiol. Rev..

[59] Martincorena I., Fowler J.C., Wabik A., Lawson A.R.J., Abascal F., Hall M.W.J., Cagan A., Murai K., Mahbubani K., Stratton M.R. (2018). Somatic mutant clones colonize the human esophagus with age. Science.

[60] Lee-Six H., Olafsson S., Ellis P., Osborne R.J., Sanders M.A., Moore L., Georgakopoulos N., Torrente F., Noorani A., Goddard M. (2019). The landscape of somatic mutation in normal colorectal epithelial cells. Nature.

[61] Kakiuchi N., Ogawa S. (2021). Clonal expansion in non-cancer tissues. Nat. Rev..

[62] Oeggerli M., Tomovska S., Schraml P., Calvano-Forte D., Schafroth S., Simon R., Gasser T., Mihatsch M.J., Sauter G. (2004). E2F3 amplification and overexpression is associated with invasive tumor growth and rapid tumor cell proliferation in urinary bladder cancer. Oncogene.

[63] Moran J.D., Kim H.H., Li Z., Moreno C.S. (2019). SOX4 regulates invasion of bladder cancer cells via repression of WNT5a. Int. J. Oncol..

[64] Voutsadakis I.A. (2021). Amplification of 8p11.23 in cancers and the role of amplicon genes. Life Sci..

[65] Qi M., Pang J., Mitsiades I., Lane A.A., Rheinbay E. (2023). Loss of chromosome Y in primary tumors. Cell.

[66] Zheng C., Zheng L., Yoo J.K., Guo H., Zhang Y., Guo X., Kang B., Hu R., Huang J.Y., Zhang Q. (2017). Landscape of Infiltrating T Cells in Liver Cancer Revealed by Single-Cell Sequencing. Cell.

[67] Federico L., McGrail D.J., Bentebibel S.E., Haymaker C., Ravelli A., Forget M.A., Karpinets T., Jiang P., Reuben A., Negrao M.V. (2022). Distinct tumor-infiltrating lymphocyte landscapes are associated with clinical outcomes in localized non-small-cell lung cancer. Ann. Oncol..

[68] Wójcik M., Juhas U., Mohammadi E., Drężek-Chyła K., Rychlicka-Buniowska E., Bruhn-Olszewska B., Davies H., Mattisson J., Chojnowska K., Olszewski P. (2023). Loss of Y in regulatory T lymphocytes in the tumor micro-environment of primary colorectal cancers and liver metastases. medRxiv.

[69] Fiala C., Diamandis E.P. (2020). Mutations in normal tissues-some diagnostic and clinical implications. BMC Med..

[70] Adashek J.J., Kato S., Lippman S.M., Kurzrock R. (2020). The paradox of cancer genes in non-malignant conditions: Implications for precision medicine. Genome Med..

