# Supplementary material for: Tumor Predisposing Post-Zygotic Chromosomal Alterations in Bladder Cancer—Insights from Histologically Normal Urothelium
Source: Cancers (Basel). 2024 Feb 27;16(5):961. doi: 10.3390/cancers16050961 (PMC10930680; doi:10.3390/cancers16050961)
Supplement: Supplementary file 1 [file cancers-16-00961-s001.zip › Fig_S1-S4_2024_01_15.pdf]

**Supplementary Figures 1-4.**

**Representative examples of manual curation of cancer precursor candidates for Stankowska et al., “Tumor predisposing post-zygotic chromosomal alterations in bladder cancer - insights from histologically normal urothelium”**

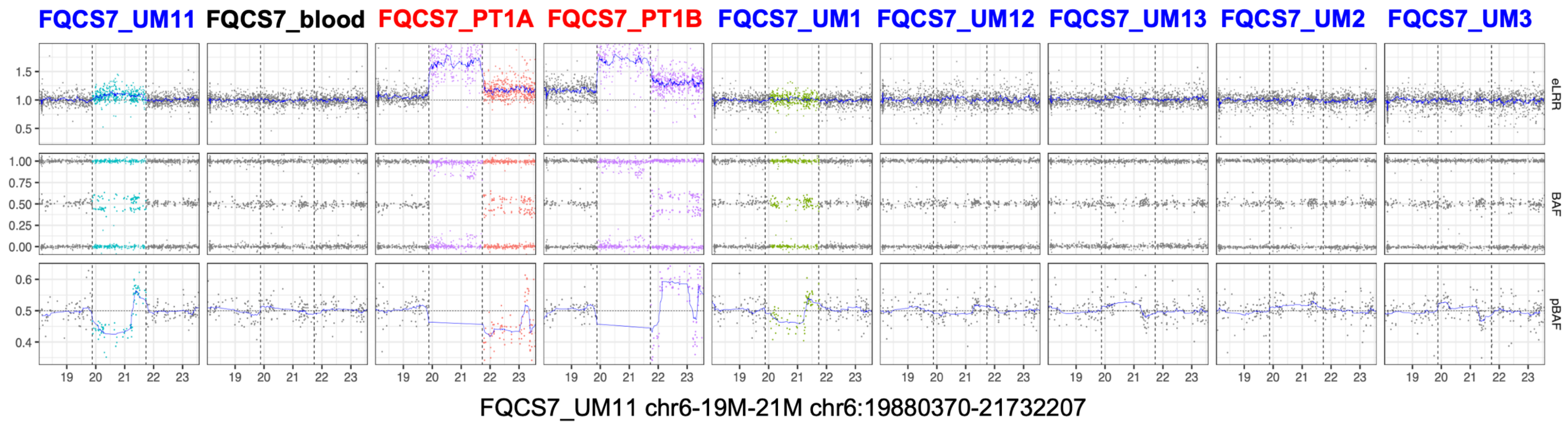


**Figure S1.** Representative example of CPC, which is a gain of chromosomal segment. Autosomal chromosomal alteration (ACA) is detected in the normal urothelium (NC) sample FQCS7_UM11, consistent with a gain of ~2 Mb on chromosome 6. The LRR, BAF and phased BAF (pBAF) plots for the ACA to be curated are shown leftmost. Genetic interval of ACA is marked by vertical dashed lines. The genotype of blood sample (FQCS7_blood) available for this patient FQCS7 is shown next to the right. Available cancerous samples (FQCS7_PT1A and FQCS7_PT1B) are plotted afterwards. Patient's remaining non-cancerous samples (FQCS7_UM1, …, FQCS7_UM3) are shown rightmost.

This alteration is also described in Supplementary Table S2 as row 242. Columns F and G show that both classifiers (see section 3.3 of the paper) agreed that this ACA is post-zygotic (PZ-ACA), which is also evident from visual examination of blood and some of non-cancerous samples (FQCS7_UM12, FQCS7_UM13, FQCS7_UM2 and FQCS7_UM3). The visual inspection confirmed this ACA and columns AP and AQ of Table S2 list it as a Gain with cellular fraction estimate of 28.6% in sample FQCS7_UM11, as required in section 3.4 of the paper. Both cancerous samples have ACA at this location (the criterion is at least 50% of length of PZ-ACA in FQCS7_UM11 overlaps with ACAs in at least one cancerous sample). After manual curation the CPC was included in further analysis and is shown in Figure 2. Please note that this aberration is present in samples FQCS7_PT1A and FQCS7_PT1B in considerably higher cellular fractions.

Note that ACA in FQCS7_UM1 failed our curation, since MoChA classified it as alteration of Undetermined type (see row 502 in Table S2).

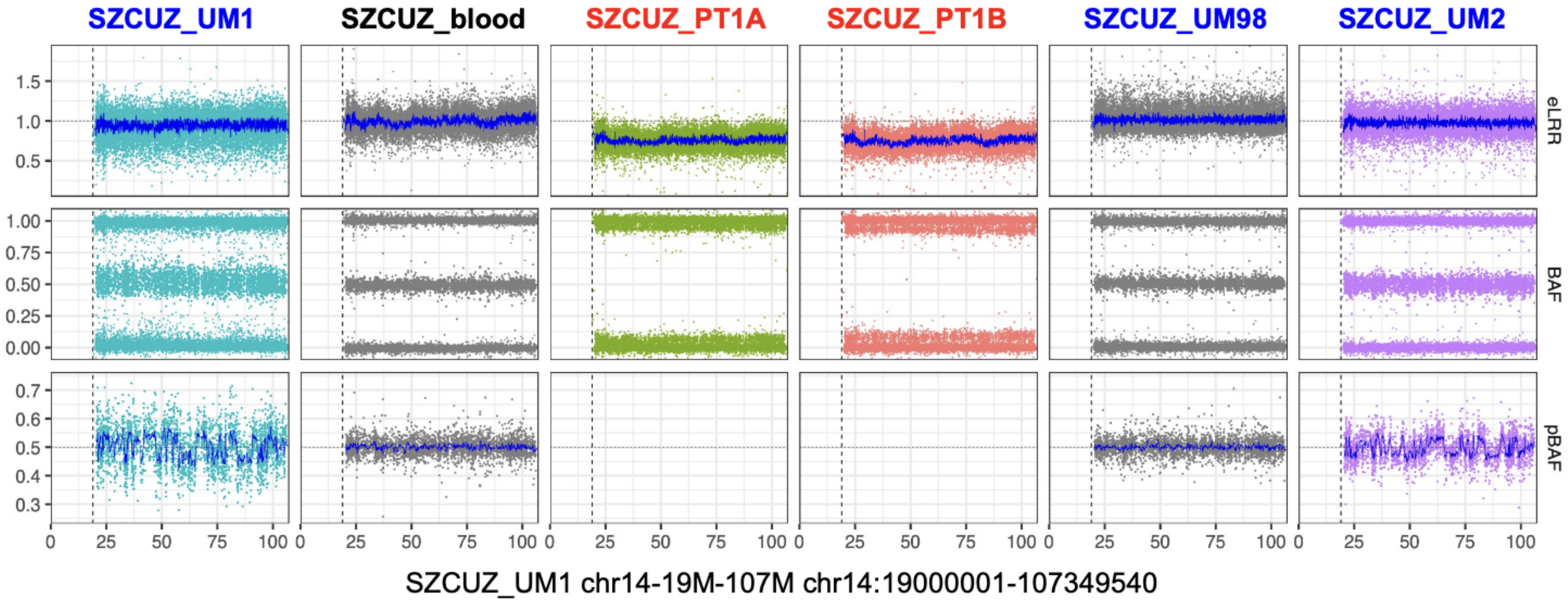


**Figure S2.** Representative example of chromosome level CPC, which is a loss of chromosome 14. Autosomal chromosomal alteration (ACA) is detected in the normal urothelium (NC) sample SZCUZ_UM1, consistent with a loss of whole chromosome 14. The LRR, BAF and phased BAF (pBAF) plots for the ACA to be curated are shown leftmost. Genetic interval of ACA is marked by vertical dashed lines. The genotype of blood sample (SZCUZ_blood) available for the patient SZCUZ is shown next to the right. Available cancerous samples (SZCUZ_PT1A and SZCUZ_PT1B) are plotted afterwards. Patient's remaining non-cancerous samples (SZCUZ_UM98 and SZCUZ_UM2) are shown rightmost.

This alteration is also described in Supplementary Table S2 as row 473. Columns F and G show that both classifiers (see section 3.3 of the paper) agreed that this ACA is post-zygotic (PZ-ACA), which is also evident from visual examination of blood and non-cancerous sample SZCUZ_UM98. The visual inspection confirmed this ACA, and columns AP and AQ of Table S2 list it as a Loss with cellular fraction estimate of 13.8% in sample SZCUZ_UM1, as required in section 3.4 of the paper. Both cancerous samples have ACA at this location (the criterion is at least 50% of length of PZ-ACA in SZCUZ_UM1 overlaps with ACAs in at least one cancerous sample). After manual curation the CPC was included in further analysis and is included in Figure 2. Please note that this aberration is present in samples SZCUZ_PT1A and SZCUZ_PT1B in considerably higher cellular fractions, essentially close to 100% of tumor cells being affected by this CPC.

Note that ACA in FQCS7_UM2 also passed our curation, see row 479 in Table S2 for details.

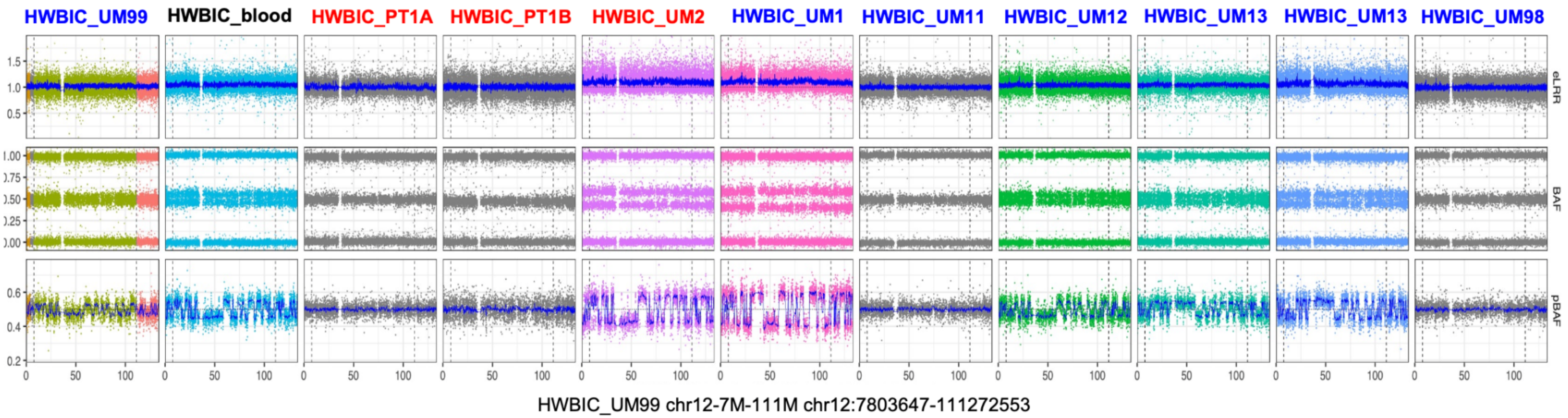


**Figure S3.** Representative example of PZ-ACA, which is not classified as CPC after manual curation. Autosomal chromosomal alteration (ACA) is detected in the normal urothelium (NC) sample HWBIC_UM99, consistent with a gain of ~103 Mb on chromosome 12. The LRR, BAF and phased BAF (pBAF) plots for the ACA to be curated are shown leftmost. Genetic interval of ACA is marked by vertical dashed lines. The genotype of blood sample (HWBIC_blood) available for this patient HWBIC is shown next to the right. Available cancerous samples (HWBIC_PT1A, HWBIC_PT1B and HWBIC_UM2) are plotted afterwards. Patient's remaining non-cancerous samples (HWBIC_UM1, …, HWBIC_UM98) are shown rightmost.

This alteration is also described in Supplementary Table S2 as row 520. Columns F and G show that both classifiers (see section 3.3 of the paper) agreed that this ACA is post-zygotic (PZ-ACA), which is also evident from visual examination of non-cancerous samples HWBIC_UM11 and HWBIC_UM98. The visual inspection confirmed this ACA, and columns AP and AQ of Table S2 list it as a Gain with cellular fraction estimate of 12% in sample HWBIC_UM99, as required in section 3.4 of the paper. A cancerous sample HWBIC_UM2 has an ACA at this location (the criterion is at least 50% of length of PZ-ACA in HWBIC_UM99 overlaps with ACAs in at least one cancerous sample). This ACA failed CPC criteria because blood sample (HWBIC_blood) also shows its presence at this location. Thus, this PZ-ACA did not qualify to be shown in Figure 2.

Note that this aberration is absent in two out of three cancerous samples (HWBIC_PT1A and HWBIC_PT1B), highlighting the complicated multi-clonal nature of BLCA.

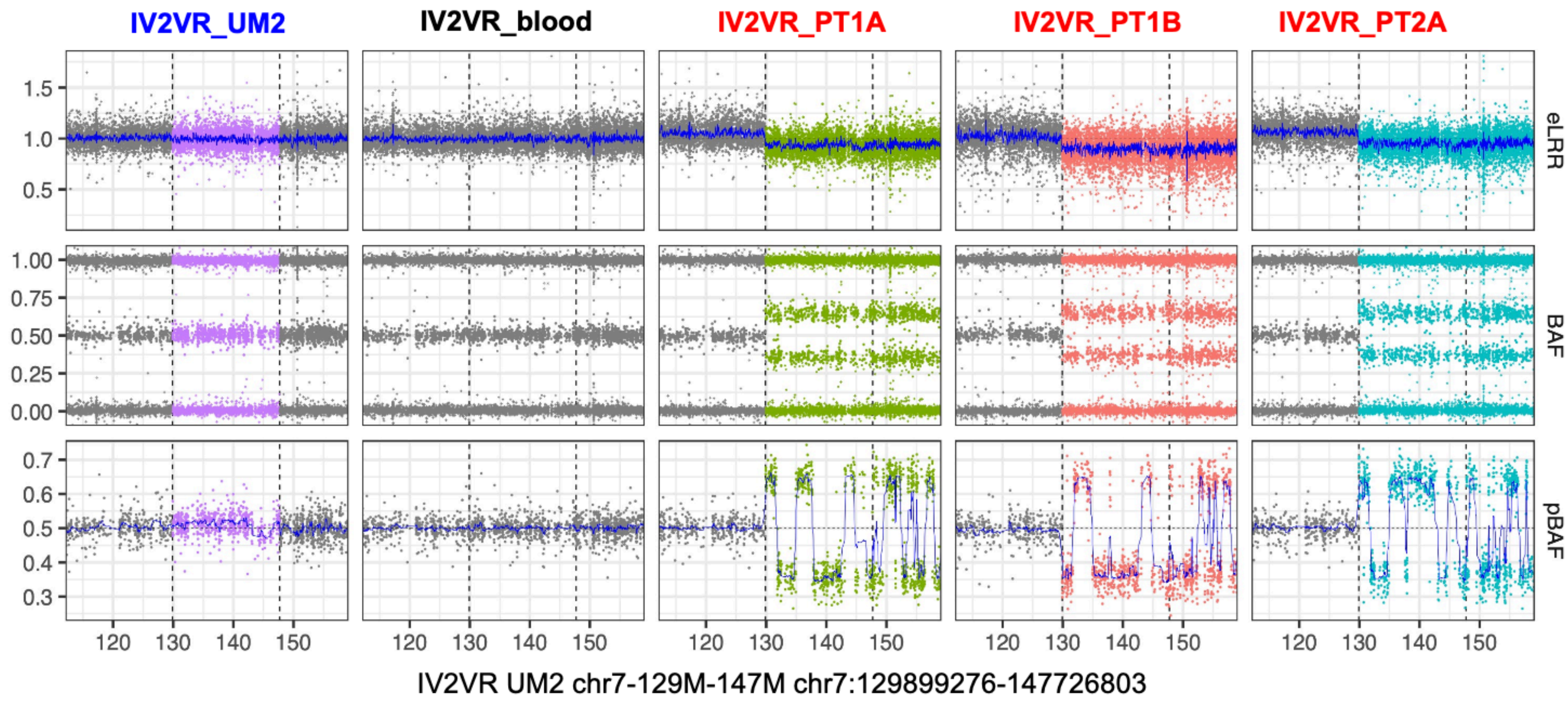


**Figure S4.** Representative example of PZ-ACA, which is not classified as CPC after manual curation. Autosomal chromosomal alteration (ACA) is detected in the normal urothelium (NC) sample IV2VR_UM2, consistent with a loss of ~18 Mb on chromosome 7. The LRR, BAF and phased BAF (pBAF) plots for the ACA to be curated are shown leftmost. Genetic interval of ACA is marked by vertical dashed lines. The genotype of blood sample (IV2VR_blood) available for this patient IV2VR is shown next to the right. Available cancerous samples (IV2VR_PT1A, IV2VR_PT1B and IV2VR_PT2A) are plotted afterwards. No more non-cancerous samples are available to show.

This alteration is also described in Supplementary Table S2 as row 507. Columns F and G show that both classifiers (see section 3.3 in the paper) agreed that this ACA is post-zygotic (PZ-ACA). However, this ACA shows borderline visual evidence in IV2VR_UM2 and the uncertain copy-number state "Undetermined" assigned by MoChA in column AP of Table S2. Thus, this PZ-ACA did not qualify to be shown in Figure 2.

Note that all three cancerous samples have ACAs at this location. This highlights the need to analyze many normal urothelium samples from the same patient whenever possible, since the evidence provided by the single available non-cancerous sample IV2VR_UM2 is borderline.
